# Endoscopic Ultrasound for Preoperative Esophageal Squamous Cell Carcinoma: a Meta-Analysis

**DOI:** 10.1371/journal.pone.0158373

**Published:** 2016-07-07

**Authors:** Lin-na Luo, Long-jun He, Xiao-yan Gao, Xin-xin Huang, Hong-bo Shan, Guang-yu Luo, Yin Li, Shi-yong Lin, Guo-bao Wang, Rong Zhang, Guo-liang Xu, Jian-jun Li

**Affiliations:** 1 Department of Endoscopy, Sun Yat-sen University Cancer Center, Guangzhou, China; 2 State Key Laboratory of Oncology in South China, Guangzhou, China; 3 Guangdong Esophageal Cancer Institute, Guangzhou, China; 4 Collaborative Innovation Center for Cancer Medicine, Guangzhou, China; University Medical Center of Princeton/Rutgers Robert Wood Johnson Medical School, UNITED STATES

## Abstract

**Background:**

Treatment options and prognosis of esophageal squamous cell carcinoma (ESCC) depend on the primary tumor depth (T-staging) and regional lymph node status (N-staging). Endoscopic ultrasound (EUS) has emerged as a useful staging tool, but studies regarding its benefits have been variable. The objective of this study was to evaluate the diagnostic accuracy of EUS for detecting preoperative ESCC.

**Methods:**

We included in our meta-analysis studies involving EUS-based staging of preoperative ESCC compared with pathological staging. Using a random-effects model, we performed a meta-analysis of the accuracy of EUS by calculating pooled estimates of sensitivity, specificity and the diagnostic odds ratio. In addition, we created a summary receiver operating characteristic (SROC) curve.

**Results:**

Forty-four studies (n = 2880) met the inclusion criteria. The pooled sensitivity and specificity of T1 were 77% (95%CI: 73 to 80) and 95% (95%CI: 94 to 96). Among the T1 patients, EUS had a pooled sensitivity in differentiating T1a and T1b of 84% (95%CI: 80 to 88) and 83% (95%CI: 80 to 86), and a specificity of 91% (95%CI: 88 to 94) and 89% (95%CI: 86 to 92). To stage T4, EUS had a pooled sensitivity of 84% (95%CI: 79 to 89) and a specificity of 96% (95%CI: 95 to 97). The overall accuracy of EUS for T-staging was 79% (95%CI: 77 to 80), and for N-staging, 71% (95%CI: 69 to 73).

**Conclusions:**

EUS has good diagnostic accuracy for staging ESCC, which has better performance in T1 sub-staging (T1a and T1b) and advanced disease (T4).

## Introduction

Esophageal cancer is one of the ten leading causes of cancer-related mortality worldwide[1]; nearly 90% of the mortality is esophageal squamous cell carcinoma (ESCC) and 70% of ESCC occurred in China. Poor outcomes in patients are usually related to diagnosis in the advanced stage and metastases in early disease[2]. Thus, early diagnosis is essential for better prognosis of ESCC.

Staging of ESCC is extremely significant in guiding treatment selection. In the early stages of the disease, it was reported that endoscopic therapy attained a complete remission of 94% with a 5-year survival of 98%[3]. Common imaging modalities used in staging preoperative ESCC include CT, MRI, PET and EUS. CT provides importance information about tumor size and regional lymph node status. However, CT alone is reported to have a sensitivity of 33% in lymph node involvement[4]. MRI has been reported to add information to the diagnosis and is equally as accurate as CT, but studies differ about how CT and MRI can contribute to a diagnosis and regarding their accuracy[5, 6]. PET’s T-staging accuracy was reported to be comparable to that of EUS and might be used clinically for staging in the future[7], but the cost of using PET is generally high. Due to the limitations of the above imaging methods, EUS emerged as a useful staging tool. EUS provides detailed information on the esophageal wall and is important in T-staging of ESCC. Furthermore, EUS has shown more capability in detecting nodal involvement than CT and MRI, with a higher sensitivity[8]. However, it is reported that the accuracy of tumor staging using EUS varies according to stages and ranges from 73% to 89%[9].

We conducted this meta-analysis to evaluate the role of EUS in the staging of ESCC. We compared the diagnostic accuracy of EUS and CT for tumor staging and detecting nodal metastasis.

## Materials and Methods

### Study population and evidence acquisition

Patients with ESCC who underwent preoperative EUS (index test) staging and had pathological staging as a reference standard were included in this study.

According to the Preferred Reporting Items for Systematic Reviews and Meta-analysis and Meta-analysis of Observational Studies in Epidemiology recommendations for study reporting[10, 11], a prospective protocol was drafted, including objectives, literature search, selection criteria, outcome measurements and methods of statistical analysis.

### Literature search

A literature search of studies in English was performed in October 2015. The search was not limited by region nor publication type. We searched PubMed, Cochrane Library, Web of Science, Embase and Google Scholar. A combination of the following MeSH subjects were employed in [Title/Abstract]: “endoscopic ultrasound/EUS”, “computed tomography/CT”, “esophageal squamous cell carcinoma/ESCC”, “tumor staging” and “nodal staging.” The related articles function was used to identify additional studies. For a series of studies investigating the same population, only the latest study with the highest state of completion was included.

### Selection criteria

Studies were included if the EUS results of preoperative patients with ESCC were confirmed by final pathological staging of surgery, endoscopic mucosal resection (EMR) or endoscopic submucosal dissection (ESD). Studies from which a 2×2 table could be completed for true-positive (TP), false-positive (FP), false-negative (FN) and true-negative (TN) values were also included. We excluded reviews, abstracts, editorials or letters, case reports and non-English publication.

### Quality assessment of articles

The methodological quality of included studies were assessed according to the Quality Assessment of Diagnostic Accuracy Studies (QUADAS-2) criteria[12], which consists of four domains: patient selection, index texts, reference standard and flow of patients through the study and timing of the index tests and reference standard (flow and timing). Each of the domains has four factors: description of patients, index tests, and reference standard and target condition; signaling questions flagging aspects of study design related to the potential for bias (yes, no or unclear); risk of bias (high, low or unclear); and concerns about applicability (high, low or unclear). If any signaling question is answered “no”, a potential for bias exists. A final result was shown by a risk map drawn by Review Manager 5.0 (Cochrane Collaboration, Oxford, UK).

### Data extraction

Two independent authors (LN Luo and LJ He) screened and reviewed all titles, abstracts and full text for eligibility and data extraction. Data were extracted when results were presented for tumor staging and nodal invasion according to the 2010 TNM classifications[13] by EUS. The data of CT study were extracted from comparison study with EUS. The absolute number of TP, FP, FN and TN test results were retrieved or calculated from the articles. We also recorded other characteristics, such as publication year, mean age of patients, proportion of males, retrospective or prospective set-up of the study and the reference standard that was used in the study. Any disagreement was resolved by the adjudicating senior author (JJ Li).

### Statistical analysis

Meta-analysis was performed for the accuracy of EUS in diagnosing preoperative ESCC. The 2×2 tables were completed for T and N-staging from each study, and 0.5 was added to the 0 value, where 0 counts occurred in at least 1 cell of the tables[14]. Based on the 2×2 tables, we calculated sensitivity, specificity, diagnostic accuracy and diagnostic odds ratio (DOR) for T and N-staging by Meta-Disc version 1.4 statistical software (Meta-Disc, Unit of Clinical Biostatistics Team of the Roman y Cajal Hospital, Madrid, Spain). All results were reported with 95% confidence intervals (CIs). Forest plots were drawn to show the point estimates in each study in relation to the pooled estimates of the summary.

A summary receiver operating characteristic curve (SROC) was constructed based on the Moses-Shapiro-Littenberg method, and the area under the curve (AUC) was a measure of the overall performance of a diagnostic test to accurately identify the condition of interest[15]. A preferred test has an AUC close to 1, and a poor test has an AUC close to 0.5[16]. The Q* index was also calculated, and it was defined as the point closest to the ideal top-left corner of the SROC space.

Heterogeneity among studies was assessed by the I^2^ statistic. Generally, an I^2^ index of 25%, 50%, and 75% represents low, moderate, and high heterogeneity[16], respectively. A random-effects model was used if heterogeneity existed; otherwise, a fixed-effects model was used.

An additional analysis was performed to compare EUS and CT for their diagnostic accuracy concerning prognosis for ESCC, using Review Manager 5.0. Forest plots were completed to screen for comparison of the summary for the pooled estimates.

## Results

**Fig 1:**
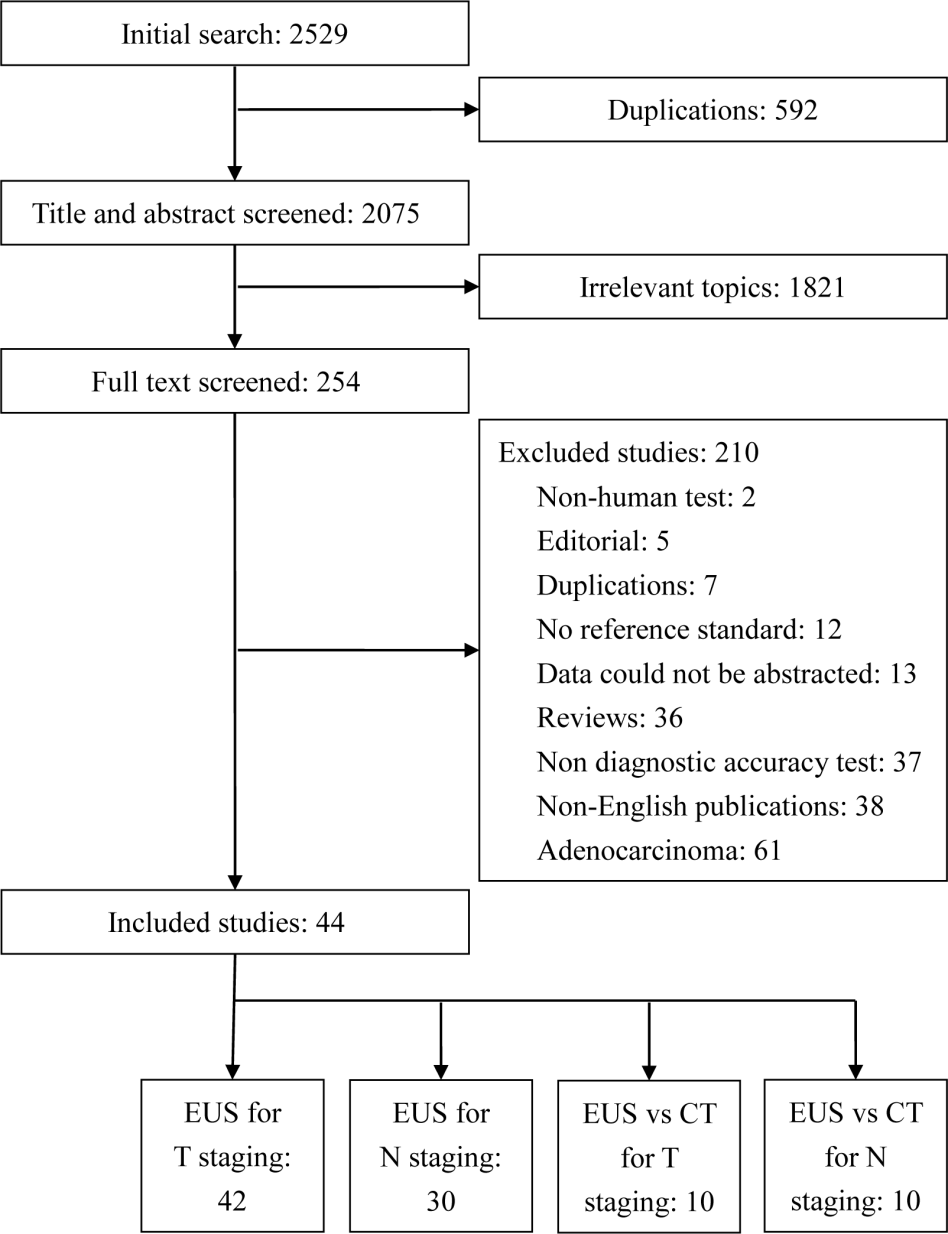
Search results.

There were 2529 studies in our initial search, of which, 254 studies were selected and reviewed. Forty-four studies[7, 8, 17–58] (n = 2880) met the inclusion criteria (Table 1), of this group, 42 studies described EUS in T-staging[7, 8, 17–35, 37–43, 45–58] and 30 described EUS in N-staging[7, 8, 17–20, 22, 26, 31, 32, 36–42, 44–47, 50–54, 56–58]. There were 9 studies[7, 25, 29, 32, 38, 48, 53, 54, 58] that reported EUS versus CT for ESCC in T-staging, and there were 10 studies[7, 8, 25, 38, 40, 44, 53, 54, 57, 58] that reported EUS versus CT for ESCC in N-staging. Most of the excluded studies were about adenocarcinoma or a non-diagnostic accuracy test (Fig 1).

**Table 1:** Characteristic of included studies.

No	Author	Year	Country	Study type	Male%	Median age	EUS frequencies MHz	EUS method	Sample size	Confirmatory test
1	Binmoeller	1995	Germany	prospective	73.6	61	7.5	radial	38	surgery
2	Catalano(End)	1994	America	retrospective	72.0	69	7.5,12	radial	100	surgery
3	Catalano(Eva)	1999	America	prospective	72.4	66	7.5,12	radial	145	surgery
4	Choi	2010	Korea	prospective	94.5	62.7	5,12,20	radial	109	surgery
5	Gheorghe	2006	Romania	prospective	92.7	61	N*	N*	41	surgery
6	Goda	2009	Malaysia	retrospective	61.4	65	20	mini-probe	101	EMR
7	Grimm	1993	America	prospective	85.7	59	7.5	radial	63	surgery
8	Hasegawa	1996	Japan	retrospective	86.4	61.5	7.5,12	radial	25	surgery
9	He	2014	China	retrospective	65.3	58	7.5,12	radial	72	surgery
10	Heintz	1991	Germany	retrospective	72.5	63	7.5,12	radial	40	surgery
11	Hunerbein.C	2003	Germany	retrospective	59.5	61	7.5,12.5	R/M ^a^	97	surgery
12	Hunerbein.M	1996	Germany	prospective	58.2	62	7.5	radial	19	surgery
13	Kawano	2003	Japan	retrospective	N*	N*	20	radial	85	surgery
14	Kienle	2002	Germany	prospective	N*	N*	7.5,12.5	radial	117	surgery
15	kutup	2007	Germany	retrospective	76.2	N*	20	radial	214	surgery
16	Lee	2014	Korea	retrospective	78.9	68.1	N*	N*	19	surgery
17	Lok	2008	China	retrospective	81.1	65.8	20	mini-probe	59	surgery
18	Massari	1997	Italy	retrospective	N*	N*	7.5,12	radial	40	surgery
19	May	2004	Germany	prospective	89.0	63.9	7.5	radial	100	S/EMR ^b^
20	Murata	1988	Japan	retrospective	N*	N*	7.5,10	radial	173	surgery
21	Murata. Y	1996	Japan	prospective	88.7	67.4	15,20	mini-probe	53	surgery
22	Natsugoe	1996	Japan	prospective	89.2	62	7.5	radial	37	surgery
23	Nesje	2000	Norway	prospective	82.3	66	7.5,12	linear/radial	68	surgery
24	Nishimaki	1999	Japan	prospective	88.4	62	7.5,12	radial	224	surgery
25	Pham	1998	Australia	prospective	71.4	67.5	N*	radial	28	surgery
26	Sandha	2008	Canada	retrospective	82.8	68	N*	N*	29	surgery
27	Shin	2014	Korea	retrospective	95.0	63	N*	radial	240	surgery
28	Shinkai	2000	Japan	retrospective	87.6	60	7.5,12,20	radial	113	S /EMR ^b^
29	Takemoto	1986	Japan	retrospective	87.5	63.4	N*	linear array	12	surgery
30	Takizawa	2009	Japan	prospective	85.1	63	5,7.5	radial	121	surgery
31	Tekola	2014	America	retrospective	89.5	65.8	7.5	radial	38	surgery
32	Tio(End)	1990	Netherland	retrospective	71.7	61	7.5,12	radial	113	surgery
33	Tio(Endo)	1989	Netherland	prospective	68.9	62	7.5,12	radial	74	surgery
34	Tio(Eso)	1989	Netherland	prospective	74.7	62	7.5,12	radial	91	surgery
35	Toh	1993	Japan	retrospective	88.5	61.3	7.5,12	radial	26	surgery
36	Vazquez	2001	America	retrospective	62.2	64	7.5,12	R/M ^a^	37	surgery
37	Vickers J	1998	England	prospective	N*	N*	7.5,12	radial	50	surgery
38	Vickers J, AD	1998	England	retrospective	N*	N*	N*	mini-probe	50	surgery
39	Wakelin	2002	England	prospective	N*	N*	7.5,12.5	radial	36	surgery
40	Wu	2003	China	retrospective	65.1	62	12,15	mini-probe	86	surgery
41	Yanai. H	1996	Japan	retrospective	64.7	64	N*	radial	16	S/EMR ^b^
42	Yen	2012	China	retrospective	96.4	60.5	N*	R/M ^a^	28	surgery
43	Yoshikane	1994	Japan	retrospective	92.9	58	7.5,12	radial	28	surgery
44	Ziegler	1991	Germany	prospective	71.2	57.5	N*	linear array	52	surgery

N* for not metioned

^a^R/M for radial/ mini-probe

^b^S/EMR for surgery/ EMR.

### Characteristics of included studies

The included studies consisted of two types of design. There were prospective designs (43%) and retrospective designs (57%). Eight studies[28–30, 32, 35, 51–53] did not report characteristics of the patients enrolled. Twelve of the studies were from Japan; 8 studies were from Germany; 5 studies were from America; 4 studies were from China; and 3 studies were from England, Korea and Netherland, respectively. There was one study from Canada, Australia, Italy, Malaysia, Norway and Romania, respectively. All the included studies used radial, linear or mini-probe EUS machines operating at 7.5, 12 or 20 Hz.

### Quality assessment of included studies

**Fig 2:**
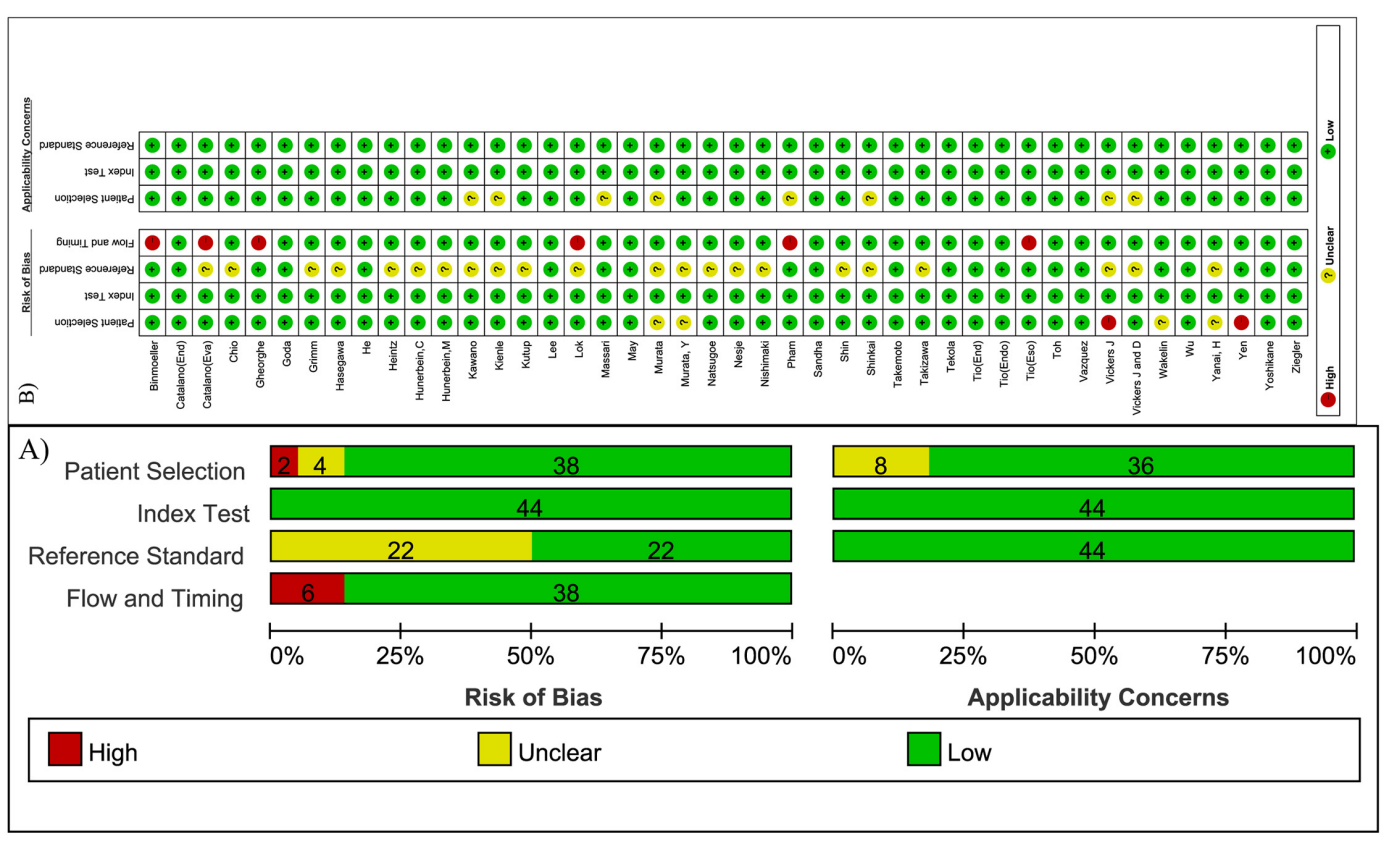
Methodological quality of included studies. A) Methodological quality graph, B) Methodological quality summary.

The included studies had a median quality score (Fig 2). Concerning the domain of patient selection bias, four studies[34, 35, 53, 55] did not definitely state that patients were consecutively or randomly enrolled, but three of the studies were of prospective design. In addition, two studies[51, 56] were case-control designed. All studies applied pathological results as the reference standard. Nevertheless, half of the studies, including eleven studies of prospective design[8, 18, 22, 26, 29, 34, 36–38, 44, 51] and eleven studies of retrospective design[23, 25, 27, 28, 30, 31, 35, 41, 42, 52, 55], did not clearly demonstrate a blind between EUS and pathological results, but biased reporting might be considered a potential explanation in these cases. For the time and flow domain, six studies[17, 18, 20, 31, 39, 47] did not include all patients in the calculations because of esophageal stenosis or pathological results were unavailable. There was not much concern about the applicability of the index test.

Overall, none of the studies included in the meta-analysis were excluded due to methodological faults.

### Primary outcomes

**Fig 3:**
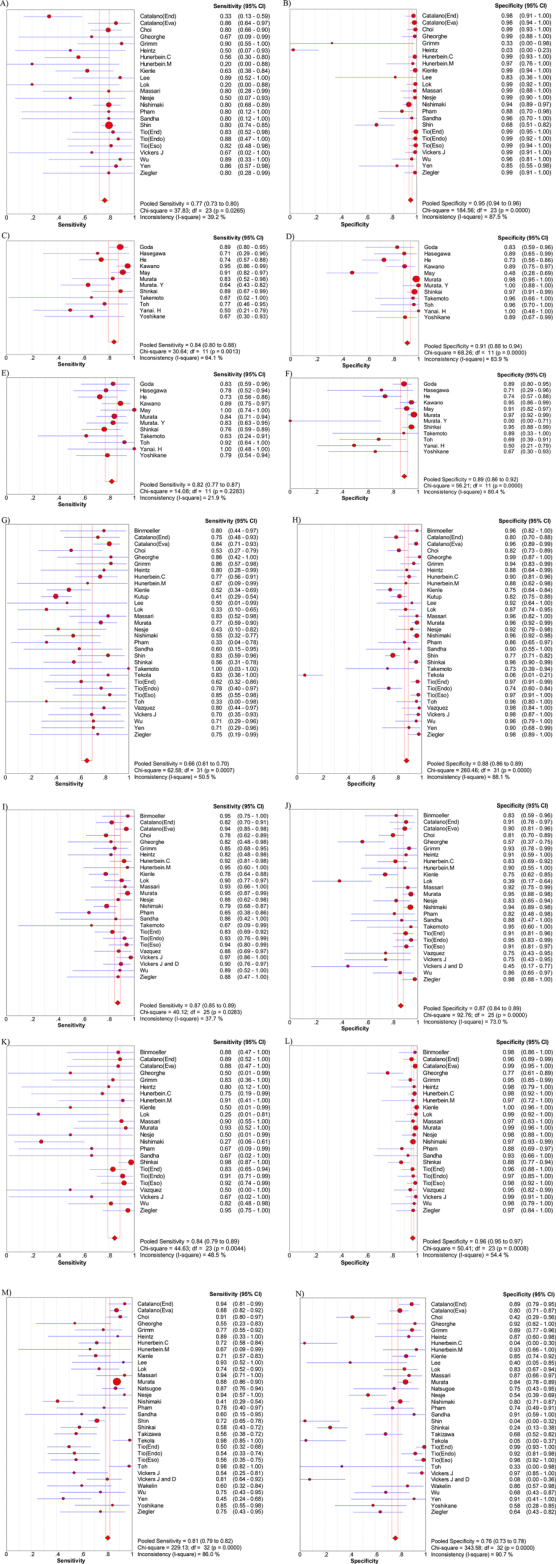
Sensitivity and specificity of EUS in staging. A), B) are sensitivity and specificity of EUS in staging T1; C), D) are sensitivity and specificity of EUS in staging T1a; E), F) are sensitivity and specificity of EUS in staging T1b; G), H) are sensitivity and specificity of EUS in staging T2; I), J) are sensitivity and specificity of EUS in staging T3; K), L) are sensitivity and specificity of EUS in staging T4; M), N) are sensitivity and specificity of EUS in staging N.

The overall T-staging diagnostic accuracy of EUS was 79% (95%CI: 77 to 80), and for the overall N-staging the diagnostic accuracy of EUS was 71% (95%CI: 69 to 73) (Table 2). Table 2 shows detailed outcomes for T and N-staging. The pooled sensitivity and specificity of EUS in diagnosing T1 stage ESCC were, respectively, 77% (95%CI: 73 to 80) and 95% (95%CI: 94 to 96) (Fig 3A and 3B). Furthermore, among this T1 group, the pooled sensitivity and specificity in differentiating T1a were, respectively, 84% (95%CI: 80 to 88), 91% (95%CI: 88 to 94) (Fig 3C and 3D), while in T1b the pooled sensitivity and specificity were, respectively, 83% (95%CI: 80% to 86) and 89% (95%CI: 86 to 92) (Fig 3E and 3F). For the T2 stage, EUS had a pooled sensitivity of 66% (95%CI: 61 to 70) and a specificity of 88% (95%CI: 86 to 89) (Fig 3G and 3H). For T3 staging cancer, EUS had a pooled sensitivity of 87% (95%CI: 85 to 89) and a pooled specificity of 87% (95%CI: 84 to 89) (Fig 3I and 3J). To diagnose T4, the sensitivity and specificity of EUS were, respectively, 84% (95%CI: 79 to 89) and 96% (95%CI: 95 to 97) (Fig 3K and 3L). To differentiate N− and N+, EUS had a pool sensitivity of 81% (95%CI: 79 to 82), and a specificity of 76% (85%CI: 73 to 78) (Fig 3M and 3N). The P value for the Chi-squared test for all the pooled estimates was <0.05.

**Table 2:** Diagnostic accuracy of EUS in T/N staging for ESCC.

	Staging	No.	Sensitivity(95%CI)	P value	Specificity(95%CI)	P value	DOR	P value	AUC (SE)	Q (SE)
**Overall accuracy**	T	42	79%* (77,80)	<0.001	-	-	-	-	-	-
	N	30	71%* (69,73)	<0.001	-	-	-	-	-	-
Staging	T1	24	77% (73,80)	<0.05	95% (94,96)	<0.001	66.43 (28.83,153.05)	<0.001	0.89(0.03)	0.82(0.03)
	T2	32	66% (61,70)	<0.001	88% (86,89)	<0.001	21.36 (12.20,37.40)	<0.001	0.83(0.04)	0.76(0.04)
	T3	26	87% (85,89)	<0.05	87% (84,89)	<0.001	42.42 (25.90,69.46)	<0.001	0.93(0.01)	0.87(0.01)
	T4	24	84% (79,89)	<0.05	96% (95,97)	<0.001	114.87 (60.86,217.46)	0.184	0.98(0.01)	0.94(0.01)
	N	34	81% (79,82)	<0.001	76% (73,78)	<0.001	9.82 (5.37,17.95)	<0.001	0.83(0.03)	0.76(0.02)
Sub-staging	T1a	12	84% (80,88)	<0.05	91% (88,94)	<0.001	39.74 (16.91,93.40)	<0.05	0.92(0.02)	0.85(0.03)
	T1b	12	83% (80,86)	<0.05	89% (86,92)	<0.001	26.97 (11.11,65.47)	<0.05	0.90(0.02)	0.83(0.02)

*overall accuracy rate of T/N staging.

**Fig 4:**
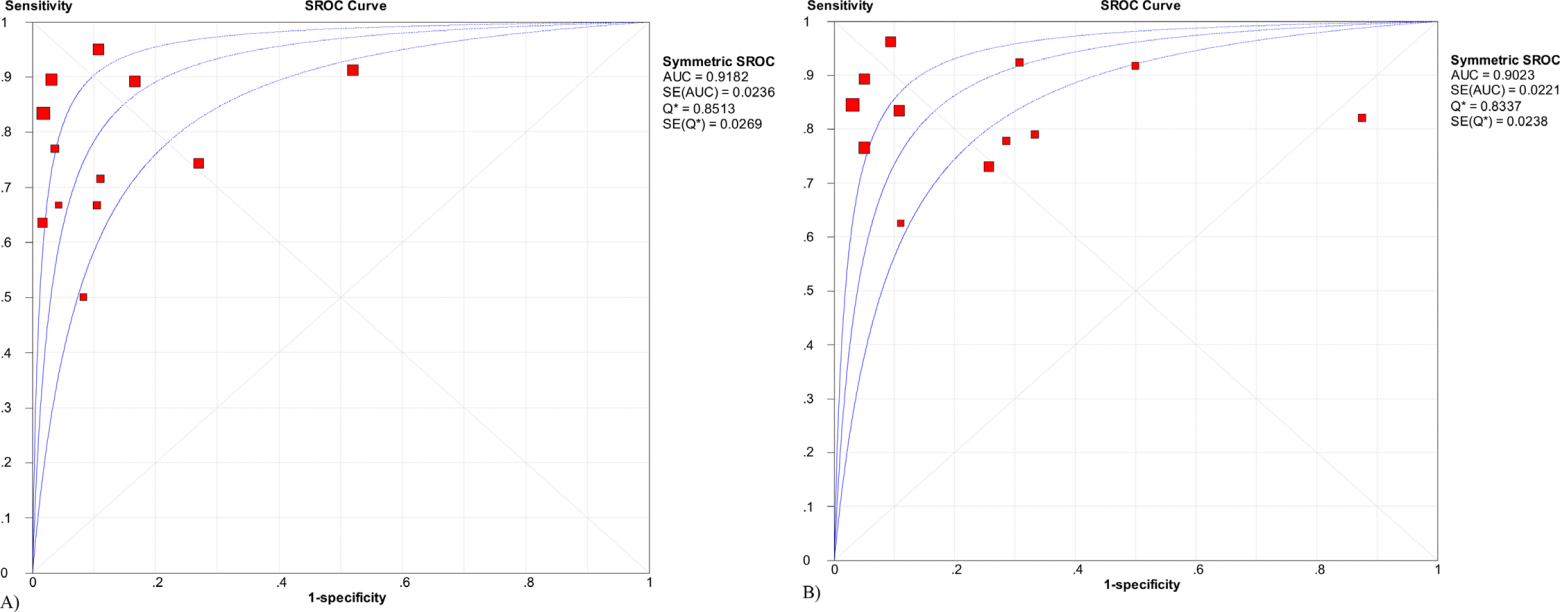
SROC curve of sub-staging for early disease. A) SROC curve for T1a; B) SROC curve for T1b.

The diagnostic odds ratios (DOR) for T1, T2, T3, and T4 were 66.43, 21.36, 42.42 and 114.87, respectively (Table 2). All the P values for the Chi-squared test were <0.05 except for T4 (*P = 0*.*184*). A summary receiver operating characteristic (SROC) curve was created for AUC and the Q* value. The drawn AUCs were 0.89, 0.83, 0.93, and 0.98 for T1, T2, T3 and T4, respectively (Table 2). To diagnose T1a and T1b, the AUC of the SROC curve was 0.92 and 0.90, respectively (Fig 4).

### Secondary outcomes

**Fig 5:**
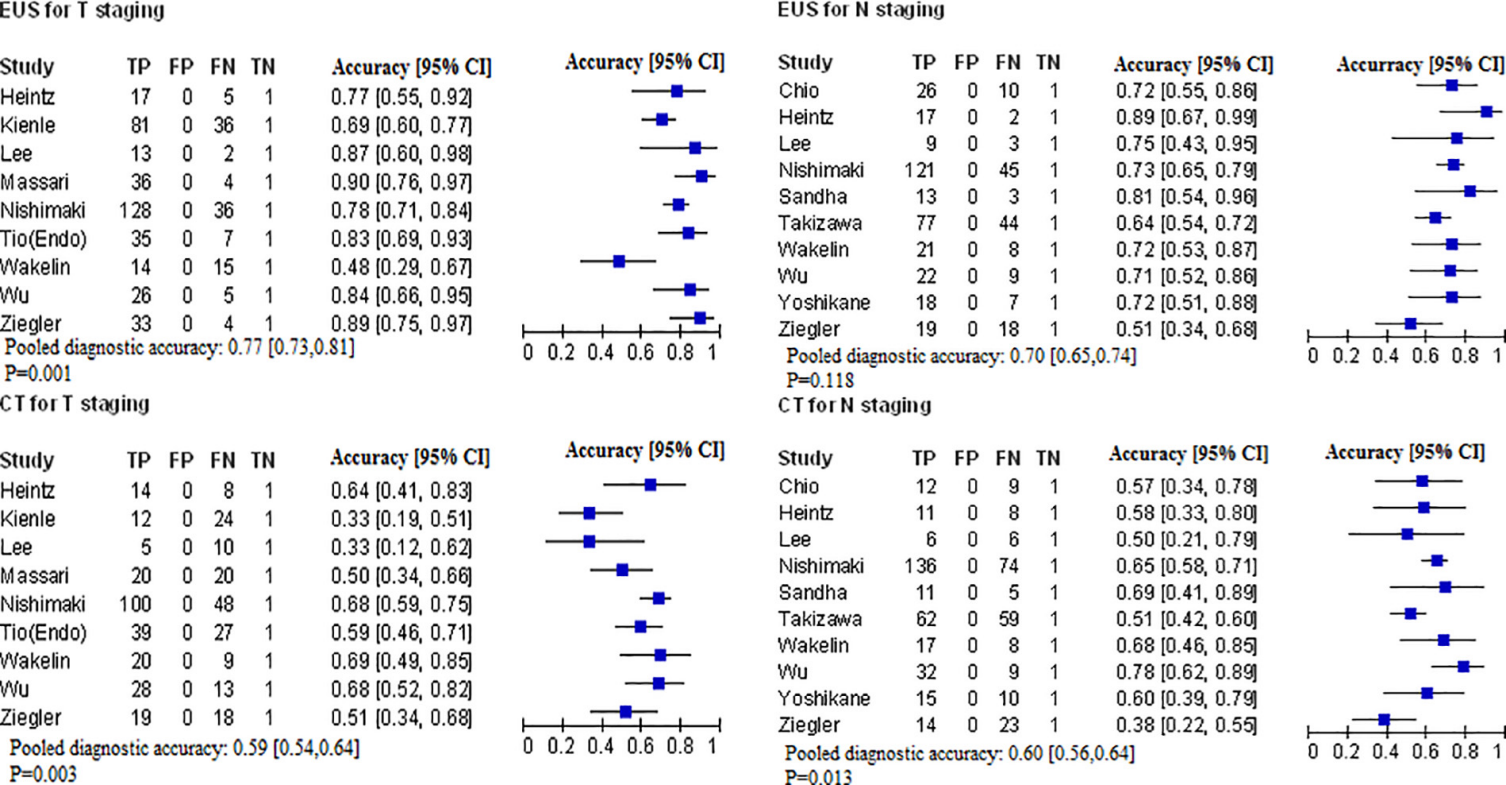
Diagnostic accuracy of EUS versus CT for ESCC.

The diagnostic accuracies of EUS and CT in T-staging were 77% (95%CI: 73% to 81%) and 59% (95%CI: 54 to 64), respectively. In N-staging, the diagnostic accuracy for EUS compared to CT was 70% (95%CI: 65 to 74) for EUS and 60% (95%CI: 56 to 64) for CT. The P value for the Chi-squared test was <0.05, except for N-staging by EUS (*P = 0*.*118*) (Fig 5).

### Effect of technology

EUS has been widely used in clinic since the late 1990s, so we divided our study into two periods of time, 1986–1999 and 2000–2014. The accuracy of EUS for T-staging and N-staging of patients with ESCC is shown in Table 3.

**Table 3:** Diagnostic accuracy of EUS in T/N staging for ESCC over 1986–2014.

	Year	T-staging		N-staging		
		Accuracy	*P*		Accuracy	*P*	
	1986–1999	0.81	*0*.*035*		0.75	*0*.*235*	
	2000–2014	0.74			0.7		
2001–2014	Developed countries	0.72	*0*.*557*		0.71	*0*.*651*	
	Developing countries	0.76			0.69		
Japan	1986–1999	0.77	*0*.*068*		0.77	*0*.*163*	
	2000–2014	0.88			0.66		

## Discussion

Our meta-analysis has shown that EUS was significantly accurate in diagnosing T-staging (overall accuracy 79%) and N-staging (overall accuracy 71%). Moreover, EUS showed a better diagnostic accuracy when compared to CT in both T and N-staging. In addition, the area under the curve (AUC) of the SROC curve drawn for all stages was close to 1, indicating EUS is an excellent diagnostic test for staging ESCC, and heterogeneity among different studies is acceptable. For T-staging, EUS had a higher diagnostic accuracy for the T4 stage than that in the T1 stage; as for sub-staging of T1, our results were excellent in differentiating T1a from the T1b stage. In addition, Thosani et al[59] conducted a meta-analysis on 19 studies regarding the sensitivity and specificity of EUS in sub-staging. They found the sensitivity of EUS for T1a was 85% and the specificity of EUS was 87%. Thosani et al further found EUS sensitivity and specificity to be 86% for T1b, and they suggested that EUS with a mini-probe with higher frequencies allowed more precise T-staging, though no significant difference was found.

With the increasing demand for detecting early and curable stages, many new technical improvements have appeared to improve EUS. For detecting early ESCC disease, high-resolution endoscopy was created for clearer vision for a shorter focal distance and can be combined with an endoscopic magnification system to perform close detailed inspection[60]. Another important development is narrow-band imaging (NBI), an optical filter technique for enhancing mucosal surface contrast for visualizing features such as vascular architecture without the use of dyes. The information about changes of submucosal vessel loops provided by NBI may not be enough to detect lesion depth, which restricts the clinical application of NBI. However, when combined with EUS, NBI can overcome the disadvantage mentioned above. Goda et al[21] suggested that magnifying endoscopy used with NBI showed advantages in staging superficial ESCC, when compared to HRE. Moreover, investigators reported that NBI had an accuracy of 85.2% for diagnosing sub-stages of early disease, and had a slightly lower accuracy rate (77.8%) for inexperienced endoscopists[21, 61]. Furthermore, the novel technique of EUS combined with submucosal saline injection improved diagnostic accuracy by more than 20%, which was better than EUS alone[62].

With regard to advanced disease, Srinivas et al[63] performed a meta-analysis on forty-nine studies and reported that EUS had a sensitivity of 92.4% and a specificity of 97.4% in diagnosing T4, which included both adenocarcinoma and squamous cell carcinoma., Srinivas et al had outcomes similar to this study. However, few studies focus on sub-staging of advanced ESCC. Differentiating advanced disease as T4a and T4b can be of benefit for treatment selection because advanced disease is resectable for patients with T4a stage and unresectable for T4b stage according to AJCC. We propose that more effort should be devoted to improving diagnostic methods of distinguishing T4a and T4b, especially using EUS and its innovations.

For N-staging, we found that EUS had good diagnostic accuracy with an AUC close to 1 (AUC = 0.83, Q* = 0.76), and its accuracy was superior to that of CT, though this difference was not statistically significant. Therefore, our results suggest that by adjusting a protocol, EUS may become a promising test for determining N-staging. However, in clinical practice, EUS has a limitation in regard to nodal invasion because the adjacent lymph nodes group may be beyond its view, which is inferior to lymphadenectomy. Physicians should be warned that EUS performance in N-staging needs to be further defined. Currently, a better option for detecting nodal metastases is to perform endoscopic ultrasonography-guided fine needle aspiration (EUS-FNA), an innovation based on EUS, which allows histological differentiation between benign and malignant lymph nodes[64]. Several studies reported that EUS-FNA was advocated for discovery of the present or absence of regional lymph node metastases. EUS-FNA did better than EUS alone, especially when the result would affect treatment decisions for patients[50, 64, 65].

As time being, the accuracy for EUS in staging patients with ESCC seems to be lower in 2001–2014 than that of the last century. Largely because only the developed countries mastered the technology of EUS in the last century, while more developing countries carried out research of EUS in recent period. For the developed countries, the accuracy of EUS in T-staging has be improved by 11% in the past 10 years, taking Japan as an example. Though the percentage of N-staging drops 11%, the accuracy for N-staging, actually, has been improved when taking EUS-FNA into account, which is developed in the recent 10 years. It is obvious that as the development of technology, developing countries have catch up with developed countries. The accuracy of EUS in both kinds of countries are similar in 2010 to 2014.

There are several limitations to our meta-analysis. First, few studies are available for evaluating outcomes of EUS in sub-staging advanced ESCC (T4). Most of the included studies enrolled patients without an advanced stage, which might be an explanation for the little data available for meta-analysis of T4a and T4b staging. Future research of sub-staging for advanced disease by the use of EUS is required. Second, there are only a limited number of studies reported for the staging accuracy of EUS for sub-staging of early disease (T1a, T1b) of ESCC. Therefore, these few studies limited the universal adequacy of our results. Third, EUS is not designed for distant metastasis (M-staging), as a diagnostic tool, so there is no such evaluation in this meta-analysis. Nevertheless, a prospective multiple-institutional trial found that FDG-PET was a promising tool to detect distant metastasis. Fourth, data for a staging comparison for EUS and CT was extracted from studies included in the meta-analysis; thus, the result might not be generalized. Using this extracted data from EUS and CT results may explain why N-staging by EUS was not statistically significant. Fifth, we mean to include studies which consisted patients before treatment, but not all studies clearly stated this point. So there is risk for us to confound the result for downstaging by mixing neoadjuvant and non-neoadujvant studies. Finally, the methodological quality of several included studies was limited. For example, two studies were prepared as case-controls, and half of the included studies did not explicitly state a blind for interpretation.

In summary, we found that EUS was highly accurate in diagnosing ESCC, especially in sub-staging primary tumor depth and advanced disease. Our data continuously discuss comparison of EUS and CT for ESCC diagnose. EUS is superior to CT in T-staging because the use of EUS allows clear recognition of esophageal wall layers. For regional lymph node status, EUS has greater accuracy than that of CT, though the difference was not statistically significant. Further research is required to improve the diagnostic accuracy of EUS for sub-staging of advanced ESCC, and refinement of staging techniques is needed for prognosis prediction and treatment selection.
